# Combined transcriptome and metabolome integrated analysis of *Acer mandshuricum* to reveal candidate genes involved in anthocyanin accumulation

**DOI:** 10.1038/s41598-021-02607-2

**Published:** 2021-11-30

**Authors:** Shikai Zhang, Wang Zhan, Anran Sun, Ying Xie, Zhiming Han, Xibin Qu, Jiayi Wang, Laifu Zhang, Mingshun Tian, Xuhong Pang, Jinbao Zhang, Xiyang Zhao

**Affiliations:** 1 State Key Laboratory of Tree Genetics and Breeding, Northeast Forestry University, Harbin, 150040 China; 2 College of Forestry and Grassland, Jilin Agricultural University, Changchun, 130118 China; 3 Baishishan Forestry Bureau, Jiaohe, 132500 China

**Keywords:** Genetics, Molecular biology, Physiology, Plant sciences

## Abstract

The red color formation of *Acer mandshuricum* leaves is caused by the accumulation of anthocyanins primarily, but the molecular mechanism researches which underlie anthocyanin biosynthesis in *A. mandshuricum* were still lacking. Therefore, we combined the transcriptome and metabolome and analyzed the regulatory mechanism and accumulation pattern of anthocyanins in three different leaf color states. In our results, 26 anthocyanins were identified. Notably, the metabolite cyanidin 3-O-glucoside was found that significantly correlated with the color formation, was the predominant metabolite in anthocyanin biosynthesis of *A. mandshuricum*. By the way, two key structural genes *ANS* (*Cluster-20561.86285*) and *BZ1* (*Cluster-20561.99238*) in anthocyanidin biosynthesis pathway were significantly up-regulated in RL, suggesting that they might enhance accumulation of cyanidin 3-O-glucoside which is their downstream metabolite, and contributed the red formation of *A. mandshuricum* leaves. Additionally, most TFs (e.g., MYBs, bZIPs and bHLHs) were detected differentially expressed in three leaf color stages that could participate in anthocyanin accumulation. This study sheds light on the anthocyanin molecular regulation of anthocyanidin biosynthesis and accumulation underlying the different leaf color change periods in *A. mandshuricum*, and it could provide basic theory and new insight for the leaf color related genetic improvement of *A. mandshuricum*.

## Introduction

**Figure 1:**
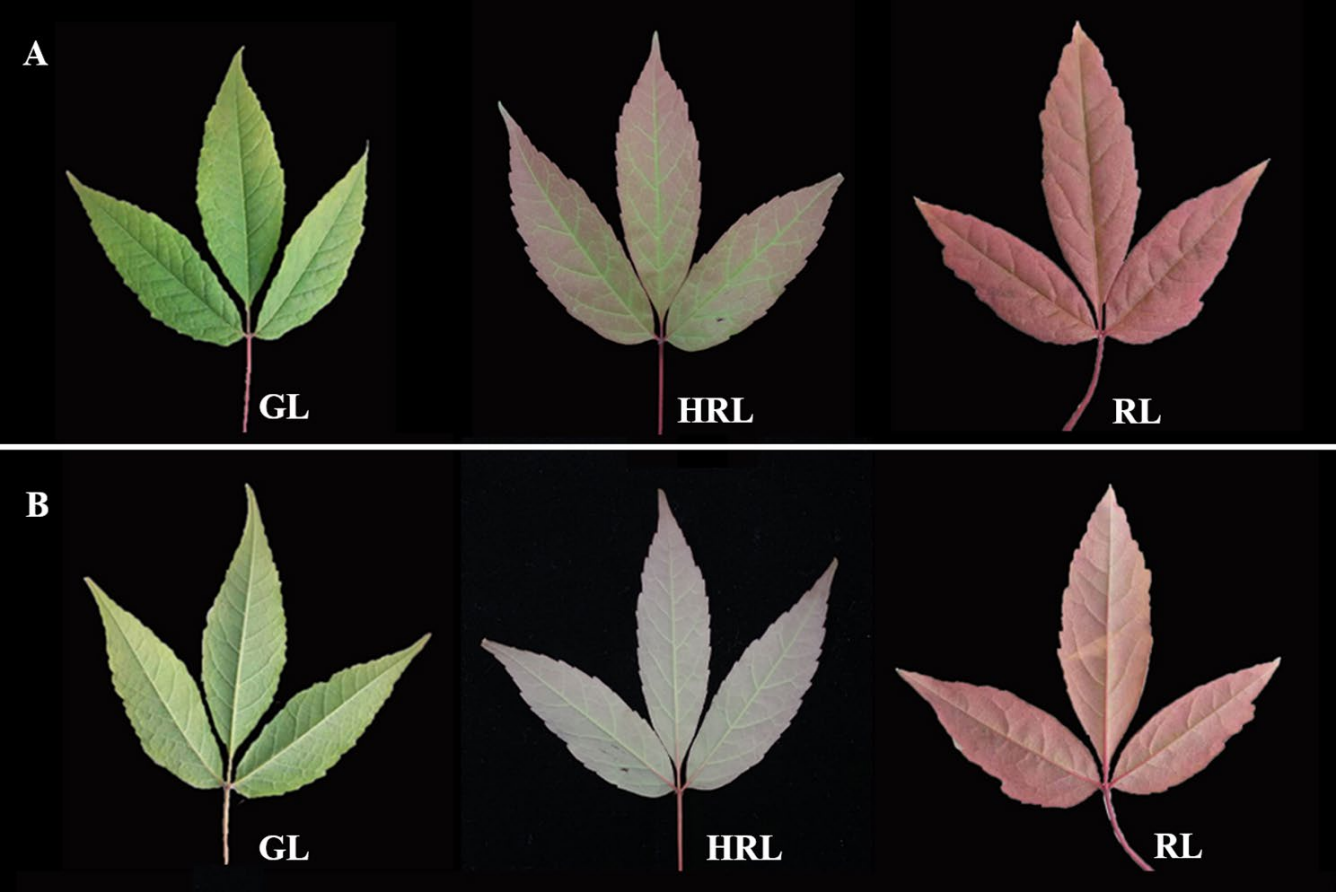
Phenotypes of *A. mandshuricum*. Green (GL), half red leaf (HRL), red leaves (RL) on front (**A**) and back (**B**).

*Acer mandshuricum*, a precious colored-leaf tree in landscapes, belongs to the genus Acer (Aceraceae) and mainly distributed among northeastern China^1^. Owing to its great ornamental value, *A. mandshuricum* has been selected as the ideal tree species for urban landscaping. It has a visually appealing tree type, and the leaves can change from green to very beautiful red in autumn (Fig. 1)^2^. With the development of the world economy, the process of urbanization is accelerating, and people's demands on the urban environment have been gradually increasing. Obviously, colored-leaf plants can play an important role in the landscape due to theircolorful leaves, strong resistance and ease of cultivation. A large number of *A. mandshuricum* trees have been transplanted into urban landscaping^3^. However, some problems have also appeared. For example, the color change period was greatly shortened when wild *A. mandshuricum* was transplanted to the city. Moreover, it was found that some genes related to colored leaves in natural forest trees could not be passed on steadily to future generations. In view of the important economic and ornamental value of *A. mandshuricum*, it is an urgent problem to study the anthocyanidin accumulation mechanism for color-related genetic improvement.

Numerous scientific studies have shown that changes in the color of leaves are caused by changes in the pigment content of their bodies, including chlorophyll, lutein, carotene, anthocyanin and more^4–6^. Among the many pigments, anthocyanins have received much attention because of their considerable ability to control leaf color changes^7^. Anthocyanin is a kind of water-soluble natural pigment widely found in plants. There are more than 20 kinds of known anthocyanins, 6 of which are more common in plants: pelargonidin, cyanidin, delphinidin, peonidin, petunidin and malvidin^8–10^. The amount of anthocyanins present can directly affect the color change of plants. At present, the synthetic pathway of anthocyanins has been elucidated in many studies, and it is a branch of the phenylpropanoid biosynthetic pathway^11,12^. The molecular mechanism of key structural genes in its pathways, such as phenylalanine ammonia-lyase (*PAL*), 4-coumarate-CoA ligase (*4CL*), chalcone synthase (*CHS*), chalcone isomerase (*CHI*), dihydroflavonol 4-reductase (*DFR*), anthocyanin synthase (*ANS*) and more, has been clarified^13–15^. At the same time, the biosynthesis of anthocyanins is also coregulated by some transcription factors, among which the greatest influences were MYBs (v-myb avian myeloblastosis viral oncogene homologs), bHLHs (basic helix-loop-helix proteins), bZIPs (basic leucine zippers) and so on^16,17^. Therefore, research on the biosynthesis process of anthocyanins in *A. mandshuricum* will help people to better interpret the molecular mechanism of leaf color change and will also contribute to genetic improvement and germplasm innovation.

In recent years, advanced sequencing technology has developed rapidly, and multiomics research has become the main means for people to explore unknown areas of cells^18,19^. Correlation analysis of the transcriptome and metabolome can realize the co-expression analysis of differential genes and differential metabolites in timing expression^20–22^. Exploring the causal relationship between genes and metabolites and combining biological function analysis such as functional annotation and enrichment of metabolic pathways is important to identify the key metabolic pathways, key genes and key metabolites^23–25^. Thus, the relationship between plant regulation mechanisms and biometric function can be analyzed systematically.

At present, the systematic study of leaf color changes and the precise positioning of leaf color-related genes in *A. mandshuricum* have not been reported. In this study, we profiled the transcriptome and metabolome changes in *A. mandshuricum* during various leaf color change stages to investigate the coloration mechanism. Our results shed light on the candidate genes and metabolic pathways underlying the leaf color change in *A. mandshuricum* and could provide fundamental theory and novel insight for the genetic improvement of *A. mandshuricum*.

## Results

### Metabolome profiling in different color period samples

**Figure 2:**
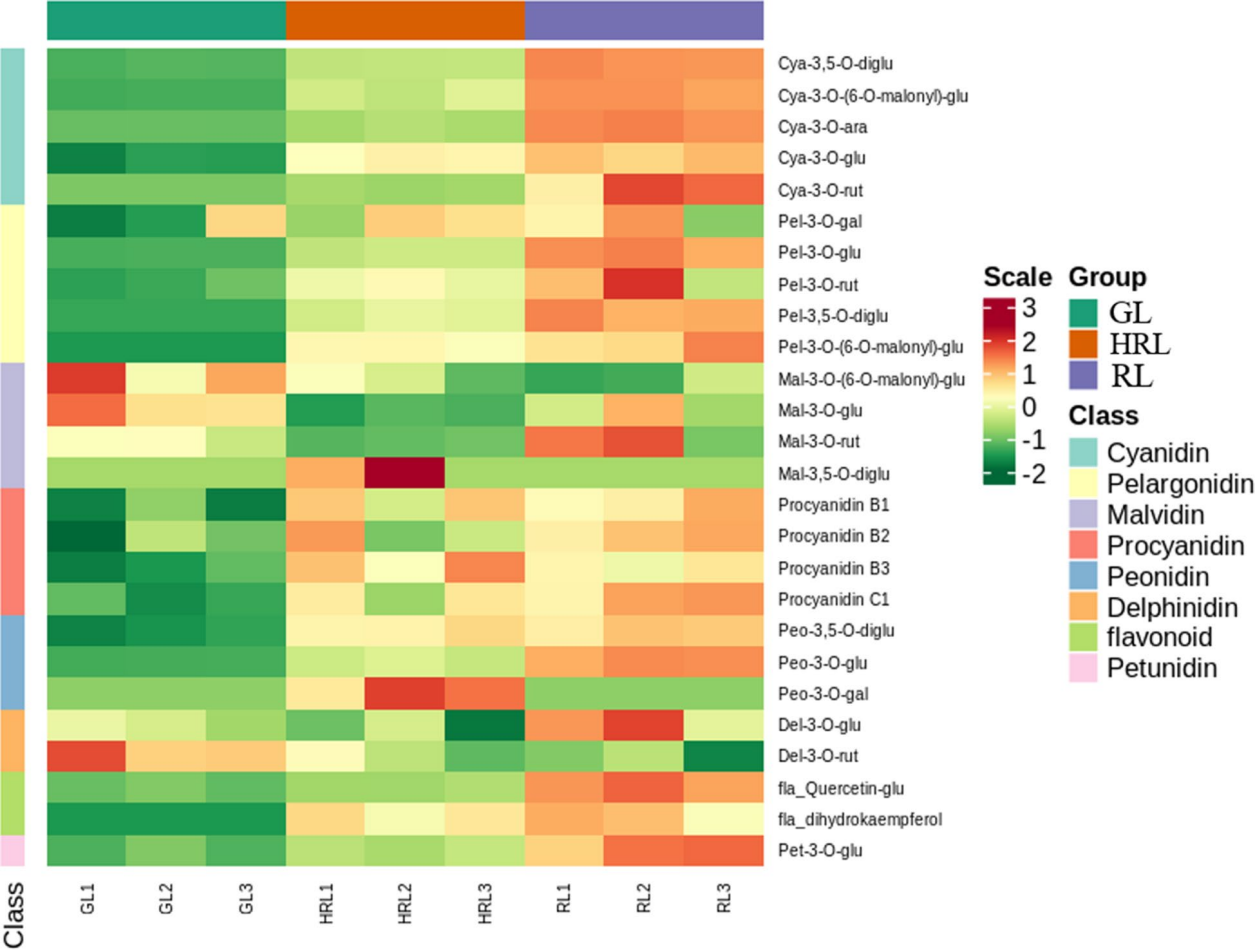
Heatmap of anthocyanidin expression level in different groups.

Throughout the leaf color change cycle of *A. mandshuricum*, it undergoes a transition from green leaves (GL) to half red leaves (HRL) and then to red leaves (RL) (Fig. 1). Therefore, to detect the change in metabolite concentration (mainly focused on anthocyanins) associated with the leaf color change process in *A. mandshuricum*, three types of leaves during different leaf color change periods were used for experiments. We profiled the metabolome of the three samples using widely targeted metabolomics approach. Then, 26 compounds involving anthocyanins were detected and grouped into 8 classes (Table S1, Fig. S1), including 5 cyanidins, 4 pelargonidins, 4 peonidins, 4 malvidins, 4 proanthocya-nidins, 2 delphinidins, 2 flavonols and 1 petunidin. Subsequently, the metabolite concentration data were used to perform a hierarchical heatmap clustering analysis of those samples. Among them, most metabolites, such as cyanidins (cyanidin 3,5-O-diglucoside, cyanidin 3-O-(6-O-malonyl-beta-d-glucoside), cyanidin 3-O-arabinoside, cyanidin 3-O-glucoside, cyanidin 3-O-rutinoside), pelargonidins (pelargonidin 3-O-glucoside, pelargonidin 3-O-(6-O-malonyl-beta-d-glucoside) and pelargonidin 3,5-O-diglucoside ), peonidins (peonidin 3,5-O-diglucoside and peonidin 3-O-glucoside), proanthocyanidins (procyanidin B1, procyanidin B2, procyanidin B3, procyanidin C1), flavonols (quercetin 3-O-glucoside, fla_dihydrokaempferol) and petunidin 3-O-glucoside were identified as significantly upregulated during the red leaf period (RL) of *A. mandshuricum* (Fig. 2). From Fig. S2, all the biological duplication groups were successfully grouped together, which indicated that the metabolic group data were highly reliable. In addition, it was obvious that the 9 samples were separated into two classes at the metabolite level, green class (GL) and deep-colored (HRL and RL) samples, and there were great differences in the patterns of metabolite accumulation between the two classes (Fig. S2).

**Figure 3:**
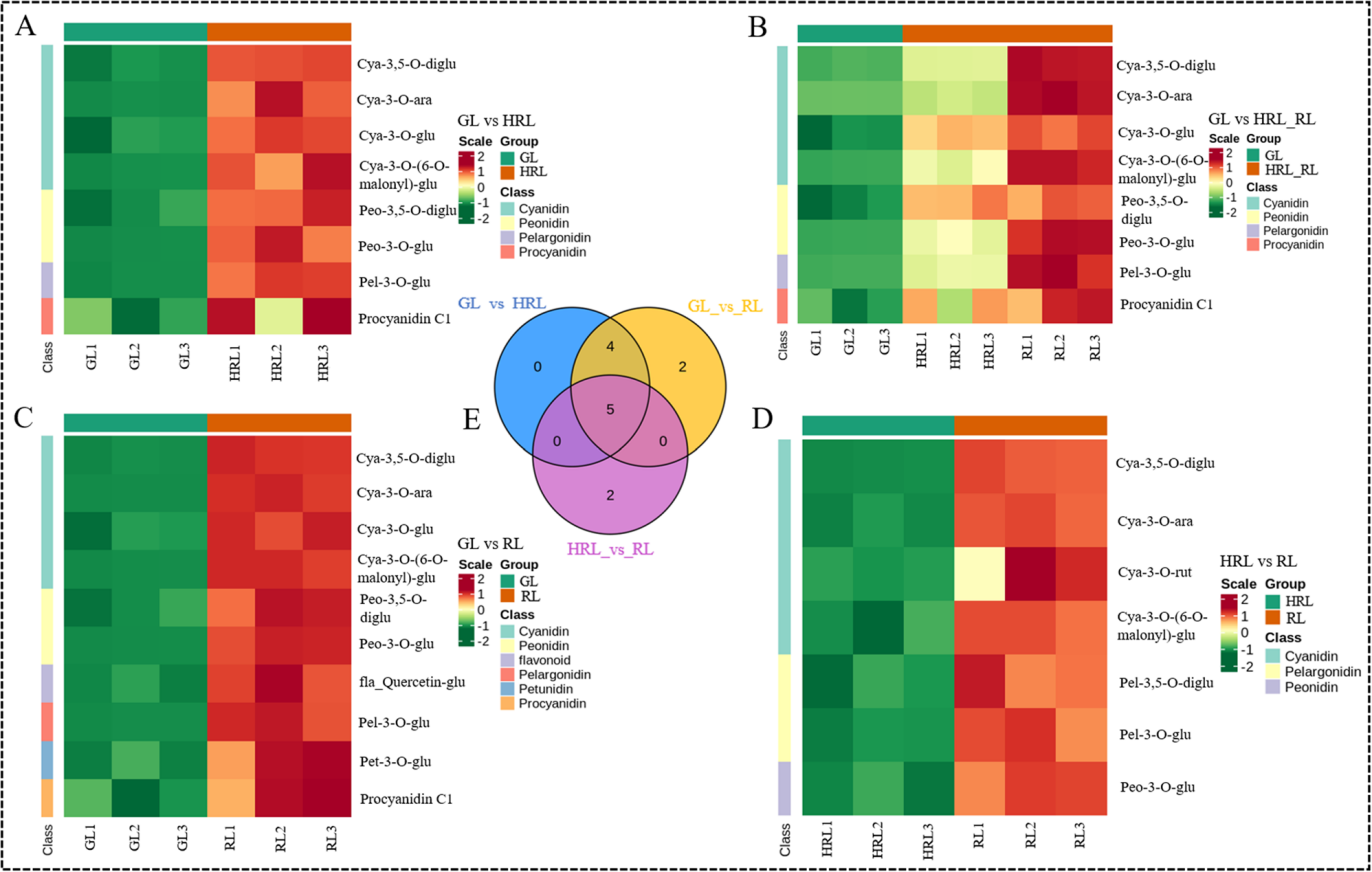
Metabolite expression level analysis of *A. mandshuricum* in different leaf sample groups. (**A**–**D**) DAMs in the anthocyanin biosynthesis pathway between four comparative groups of samples of *A. mandshuricum*. These DAMs were analyzed by KEGG enrichment analysis (**C**, GL vs HRL; **D**, GL vs HRL_RL; E, GL vs RL; F, HRL vs RL). (**E**) Venn diagram of metabolites between three compared groups of leaf samples.

To identify differentially accumulated metabolites (DAMs) between the different samples (GL vs HRL, GL vs HRL_RL, GL vs RL, HRL vs RL) in *A. mandshuricum*, the metabolites with VIP ≥ 1 and fold change ≥ 2 or ≤ 0.5 were selected, and the heatmap and Venn diagram were used to analyze the differences and expression levels of different metabolites more intuitively and quickly. The results showed that there were significant differences in metabolites between the groups in the three leaf color changes, including 8 (8 up-regulated), 8 (8 up-regulated), 10 (10 up-regulated) and 7 (7 up-regulated) DAMs in GL vs HRL, GL vs HRL, GL vs HRL-RL, GL vs RL, and HRL vs RL, respectively (Table S2). It is worth mentioning that DAMs in the anthocyanin biosynthesis pathway of the four comparative groups were significantly increased (Fig. 3A–D), so we proposed that they were related to the leaf color changes of *A. mandshuricum*, especially cyanidin 3,5-O-diglucoside, cyanidin 3-O-(6-O-malonyl-beta-d-glucoside), cyanidin 3-O-arabinoside, cyanidin 3-O-glucoside, pelargonidin 3-O-glucoside, peonidin 3-O-glucoside, and petunidin 3-O-glucoside. In the Venn diagram (Fig. 3E), 5 anthocyanins were the common metabolites among green-colored and deep-colored samples, and all of them were upregulated and enriched significantly in the anthocyanin biosynthesis pathway (ko00942) (Table S3). Therefore, we speculated that those DAMs produced in the biosynthetic pathways of anthocyanins might be key metabolites during the leaf color change periods of *A. mandshuricum*.

### Analysis of transcriptome data

Throughout the leaf color change cycle of *A. mandshuricum*, Illumina HiSeq 2000 was used to construct and sequence nine cDNA libraries based on the total RNAs of green (GL), half-red (HRL) and red (RL) leaf samples of *A. mandshuricum*. A total of 378,613,872 raw reads and 368,399,930 clean reads were obtained, and in the clean reads, the average Q20, Q30 and GC content was 97.63%, 93.42% and 43.89%, respectively (Table S4). Then, gene function annotation was carried out, and we used Blast software^26^ to compare unigenes to the KEGG (Kyoto Encyclopedia of Genes and Genomes), NR (Non-Redundant Protein Sequence Database), Swiss-Prot (a manually annotated and reviewed protein sequence database), GO (Gene Ontology), KOG (euKaryotic Ortholog Groups), Pfam databases (protein family) and Trembl. A total of 201,642 unigenes were successfully annotated, of which 150,946 unigenes (74.86%) were annotated in at least one database above (Table S5 and Fig. S3). Among the 20,162 unigenes, 96,552 (47.88%) unigenes were grouped into 25 KOG classifications; the most highly represented was group R (general function prediction only), and group C (energy production and conversion), group J (translation, ribosomal structure and biogenesis), group O (posttranslational modification, protein turnover, chaperones) and T (signal transduction mechanisms) also shared a high percentage of genes (Fig. S4). A total of 116,336 unigenes were categorized into three GO categories (biological process, cellular component and molecular function) and further divided into 60 major functional groups (Fig. S5). To reflect the expression level of transcripts, FPKM was used as an indicator to measure the level of gene expression. Principal component analysis (PCA) of the samples showed that the biological replications of GL samples and RL samples were obviously clustered together and significantly separated from each other, but the three HRL samples were dispersed (Fig. S6). Similar to the metabolomic analysis, there was a significant separation of samples with different colors, indicating that changes in the accumulation of metabolites from green to red leaves were closely controlled by differences in gene expression.

### Differentially expressed genes between different colored leaves

**Figure 4:**
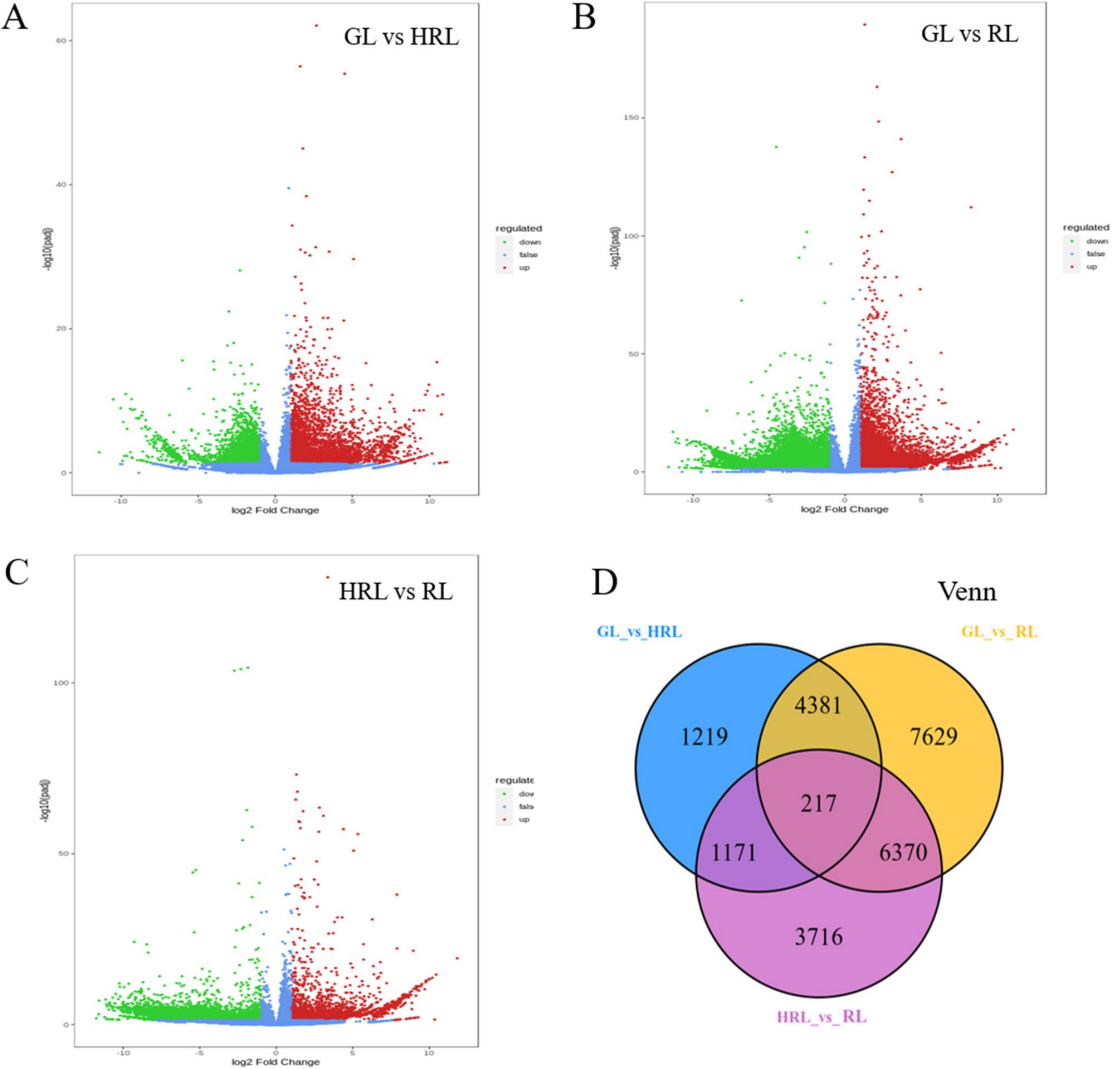
Differentially expressed genes between different colored leaves of *A. mandshuricum*. (**A**–**C**) Volcano plots were used to display the down-regulated, no-regulated and up-regulated genes between GL vs HRL, GL vs RL and HRL vs RL. (**D**) Venn map showing the common and respective DEGs between the three compared groups of leaf samples.

DESeq2^27^ was used to analyze the differential expression between different sample groups, and the results showed that a total of 6988 (3106 down-regulated and 3882 up-regulated), 18,597 (11,442 down-regulated and 7155 up-regulated), and 11,474 (9433 down-regulated and 2041 up-regulated) DEGs were obtained in the GL vs HRL, GL vs RL, and HRL vs RL groups, respectively (Table S6). The differentially expressed genes (DEGs) were identified, and the results are shown in Fig. 4. From the Venn diagram, 217 genes were the common DEGs in all comparison groups, suggesting that those genes across the three comparison groups might be associated with the leaf color change of *A. mandshuricum* (Fig. 4D). Next, we performed GO and KEGG functional annotation and pathway analysis in the three groups and found that the top enriched KEGG terms in the GL vs HRL group were ko01100 (metabolic pathways), ko01110 (biosynthesis of secondary metabolites), ko03010 (ribosome), ko04626 (plant-pathogen interaction) and ko04075 (plant hormone signal transduction) (Fig. S7). Among them, the metabolic pathways and biosynthesis of secondary metabolites were also abundant in the GL vs RL and HRL vs RL groups, indicating that these DEGs might play a key role in leaf color change pathways (Table S7). GO functional enrichment analysis of DEGs between GL vs RL and GL vs HRL revealed that flavonoid metabolic process (GO: 0009812) and flavonoid biosynthetic process (GO:0009813) were significantly enriched (Fig. S8).

**Figure 5:**
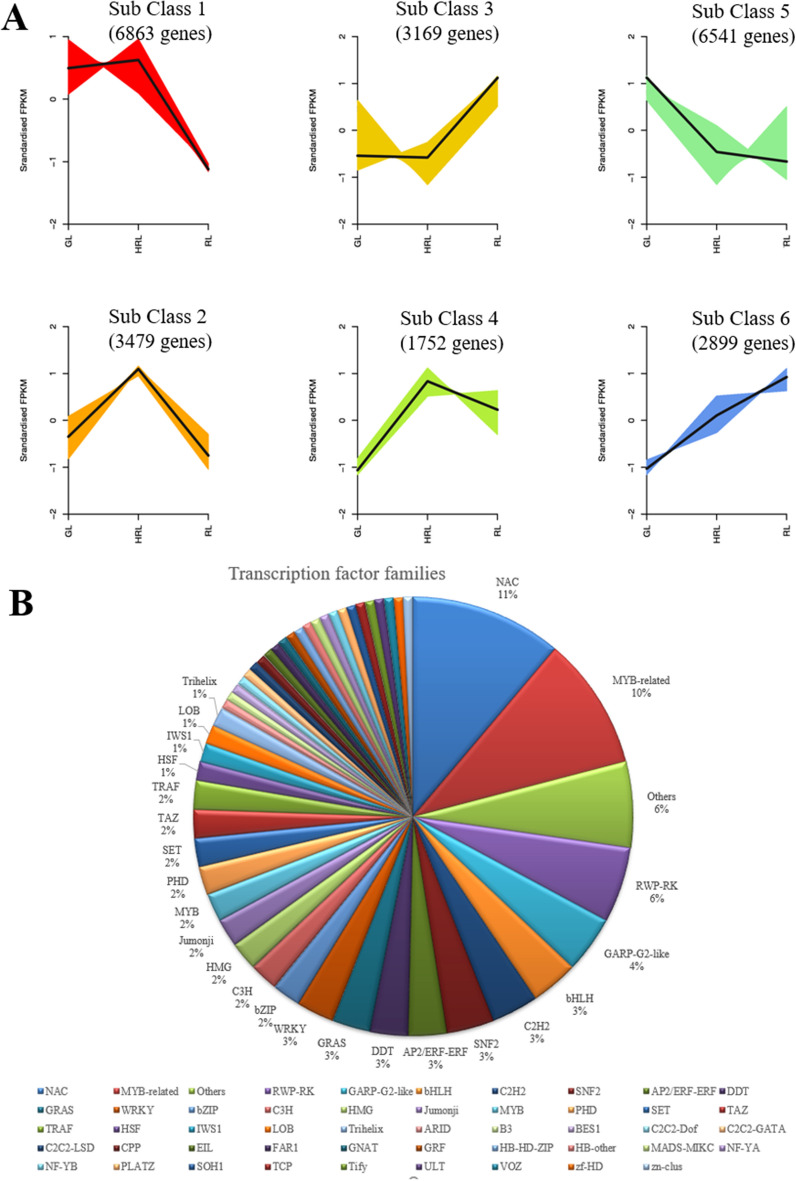
Six classes of co-expressed genes and their kinetic patterns. (**A**) Six classes of co-expressed genes. (**B**) Transcription factors (TFs) were encoded by these genes in class 6.

To gain insight into the dynamism of gene expression pattern changes over the leaf color change periods, k-means cluster analysis was performed using the FPKM value of GL, HRL, and RL transcriptome data. Six clusters of co-expressed genes with different expression trends during leaves of different colors were separated by cluster analysis, implying that each cluster may have similar molecular functions (Fig. 5A). Class 1 consisted of 6863 genes, and the expression trend first increased slowly and then decreased rapidly from green to red leaves. By contrast, class 3 contained 3169 genes with an opposite trend compared with class 1. Class 2 encompassed 3479 genes displaying an increase in expression levels from GL to HRL, but a decrease from HRL to RL; this trend was similar to class 4 (1752 genes). A total of 6541 genes were identified in class 5, displaying weak decreases from GL to RL. The most special gene was class 6, which had 2899 genes and exhibited a significant increasing trend in expression level, indicating that they were positively associated with the color change from green to red in *A. mandshuricum*. Several transcription factors (TFs) were encoded by these genes in class 6, predominantly NAC, MYB-related, RWP-RK, GARP-G2-like, bHLH and C2H2, which might play significant roles in the regulation of gene expression levels involved in leaf coloration of *A. mandshuricum* (Fig. 5B).

### Modulation of phenylpropanoid, flavonoid, and anthocyanidin biosynthesis pathway genes during leaf color change

**Figure 6:**
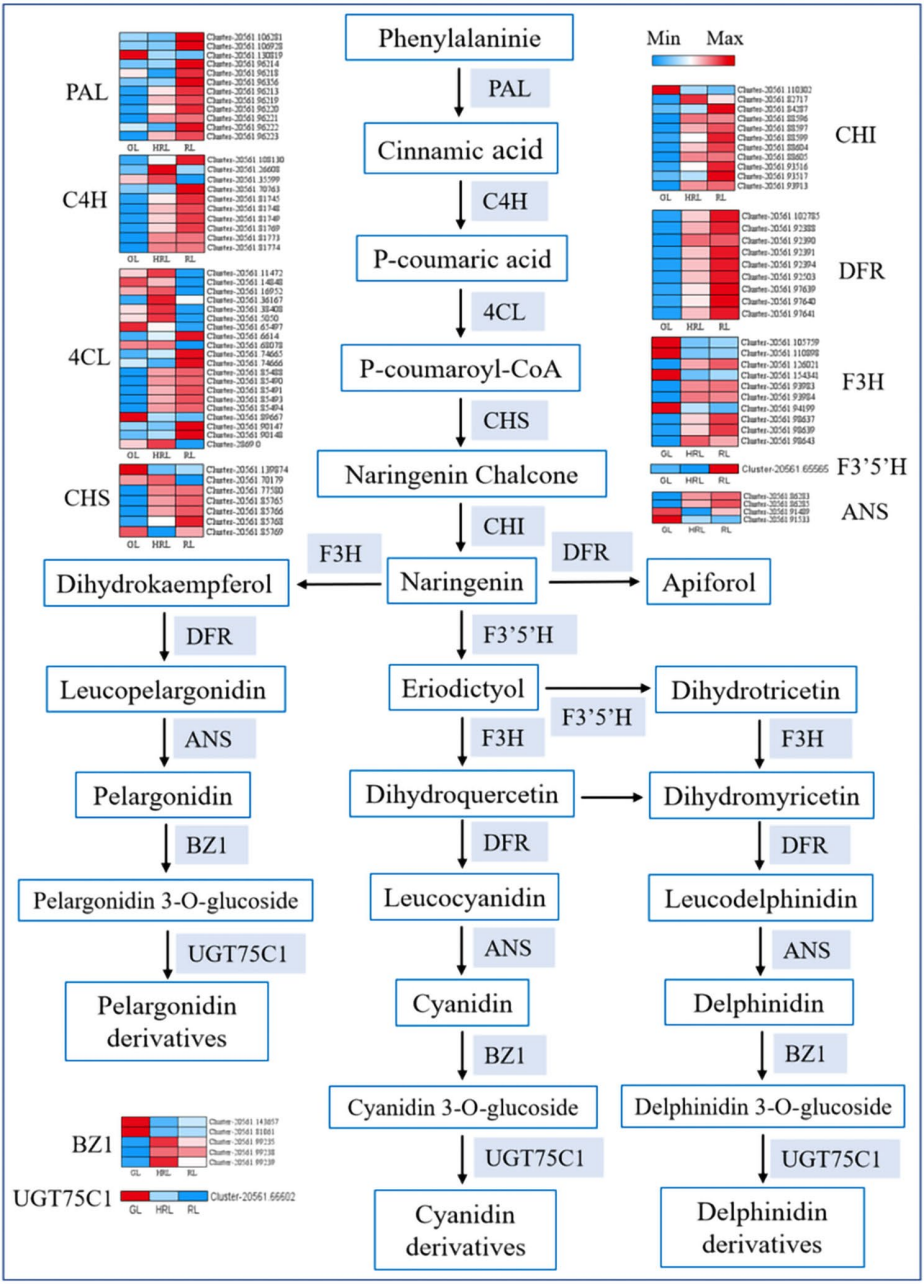
Modulation of phenylpropanoid, flavonoid, and anthocyanidin biosynthesis pathway genes during leaf color change in *A. mandshuricum*. Reconstruction of the phenylpropanoid, flavonoid and anthocyanidin biosynthetic pathways with differentially expressed structural genes. The heat map displays down-regulated and up-regulated structural genes.

Anthocyanin components determine the color of *A. mandshuricum* leaves. However, the key genes involved in *A. mandshuricum* anthocyanin synthesis have not been reported. In this study, we constructed phenylpropanoid, flavonoid, and anthocyanidin biosynthesis pathways in *A. mandshuricum*. Ninety DEGs were searched based on enrichment analysis (Table S8) and predicted the molecular mechanisms leading to red leaf coloration (Fig. 6).

From the expression levels, most key pathway genes exhibited significant changes. Indeed, encoding *PAL* (11 DEGs), *C4H* (8 DEGs), *4CL* (10 DEGs), *CHS* (4 DEGs), *CHI* (9 DEGs), *DFR* (9 DEGs), *F3H* (6 DEGs), *F3’5’H* (1 DEG), *ANS* (2 DEGs) and *BZ1* (2 DEGs) enzymes were more highly expressed in HRL and RL than in GL, denoting that those DEGs were the key genes involved in the red pigmentation of *A. mandshuricum* leaves. Conversely, the expression level of one *PAL* gene (*Cluster-20561.130819*) was significantly down-regulated from the phenylalanine to cinnamic acid route when the leaves changed from green to red. Similarly, two *4CL* genes (*Cluster-20561.65497* and *Cluster-20561.89667*), one *CHS* gene (*Cluster-20561.139874*), one *CHI* gene (*Cluster-20561.110302*), four *F3H* genes (*Cluster-20561.105759*, *Cluster-20561.110898*, *Cluster-20564.154341* and *Cluster-20561.94199*), one *ANS* gene (*Cluster-20561.91533*), two *BZ1* genes (*Cluster-20561.143.657* and *Cluster-20561.81061*) and one *UGT75C1* gene (*Cluster-20561.66602*) were also significantly down-regulated in their key pathways, implying that silencing these genes was essential for the leaf change to red. In the meantime, some notable genes were also discovered, two *C4H* genes (*Cluster-20561.26608* and *Cluster-20561.35599*), one *4CL* gene (*Cluster-20561.36167*) and one *BZ1* gene (*Cluster-20561.99239*) were activated in HRL stages, but with low expression in GL and RL. The discovery of these key differentially-expressed genes suggested that they could play a pivotal role in the process of *A. mandshuricum* leaf discoloration.

**Figure 7:**
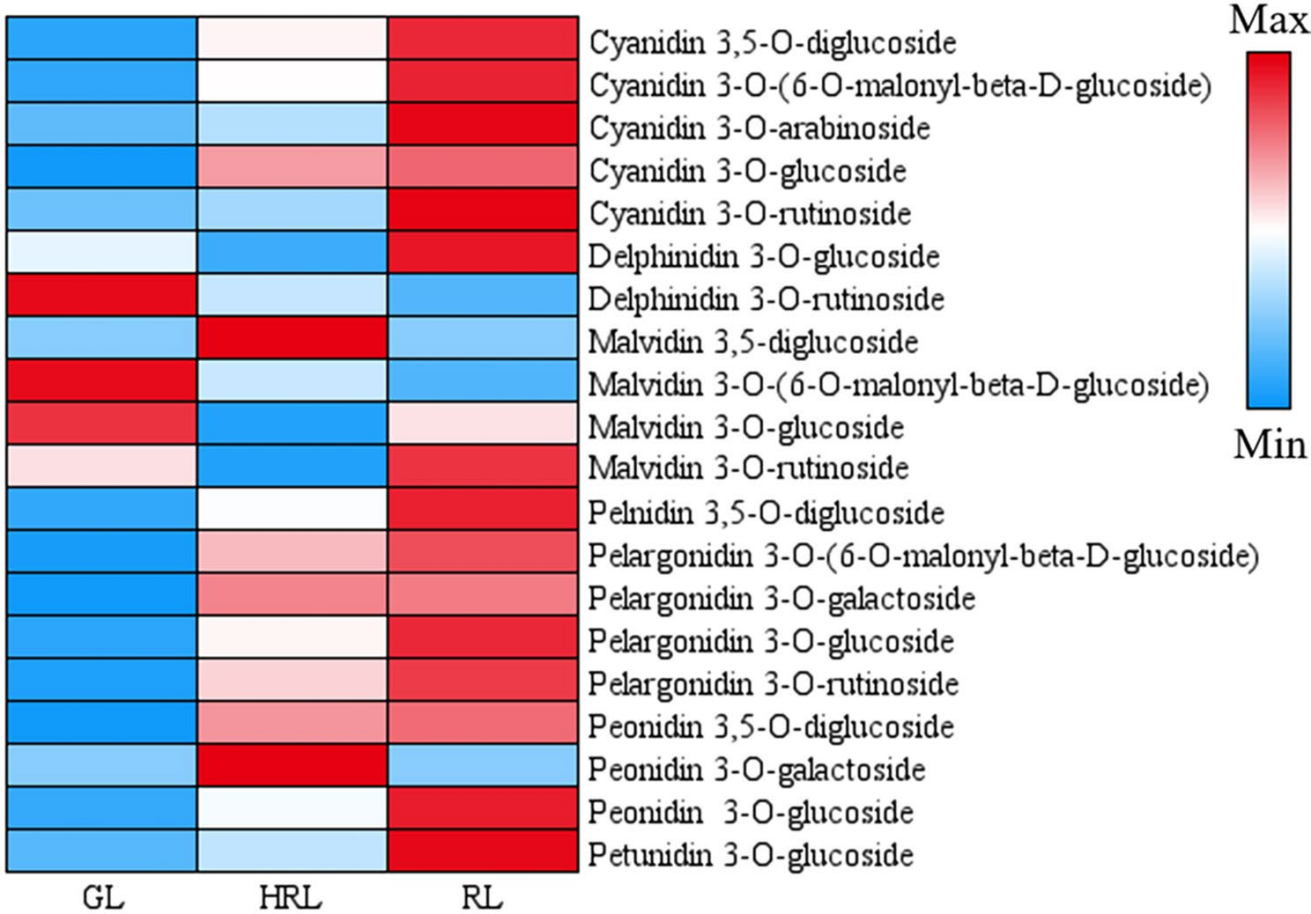
Metabolite accumulation level in anthocyanin synthesis pathways.

Comparing metabolite accumulation in anthocyanin synthesis pathways (ko00941) (Fig. 7), five cyanidins (up-regulated), two delphinidins (one up-regulated and one down-regulated), four malvidins (two up-regulated and two down-regulated), one pelnidin (up-regulated), four pelargonidins (up-regulated), and four peonidins (up-regulated) were determined. Thus, in summary, our results confirmed that the key genes identified in the biosynthesis pathways of flavonoids and anthocyanins could play a key role in *A. mandshuricum* leaves changing to red. Existing research has confirmed that MYB-related MYB, bHLH, bZIP and other transcription factors (TFs) are widely involved in the regulation of phenyl propane metabolic pathways as regulatory proteins^28,29^. Analysis of the expression levels of TFs such as bHLH, bZIP and MYB showed that some of them were down-regulated over the red periods, whereas others were significantly up-regulated, implying that these TFs positively regulate flavonoid and anthocyanidin biosynthesis pathway genes (Table S9). Overall, these TFs bHLH, bZIP, MYB and more coordinately regulate those key genes, leading to the accumulation of anthocyanin metabolites (cyanidin 3-O-arabinoside, cyanidin 3,5-O-diglucoside, cyanidin 3-O-glucoside, cyanidin 3-O-rutinoside, cyanidin 3-O-(6-O-malonyl-beta-d-glucoside), delphinidin 3-O-glucoside, malvidin 3,5-diglucoside, malvidin 3-O-rutinoside, pelnidin 3,5-O-diglucoside, pelargonidin 3-O-(6-O-malonyl-beta-d-glucoside), pelargonidin 3-O-galactoside, pelargonidin 3-O-glucoside, pelargonidin 3-O-rutinoside, peonidin 3,5-O-diglucoside, peonidin 3-O-galactoside, peonidin 3-O-glucoside, petunidin 3-O-glucoside), giving rise to the beautiful red coloration of *A. mandshuricum* leaves.

### Analysis of transcriptome factors

In the biosynthesis process of anthocyanins, it is coregulated by many transcription factors^30,31^. In this study, we listed anthocyanidin biosynthesis-related TFs in *A. mandshuricum*, and a total of 341 (106 up-regulated and 235 down-regulated), 454 (103 up-regulated and 351 down-regulated) and 981 (346 up-regulated and 635 down-regulated) differentially expressed TFs were identified in GL vs HRL, HRL vs RL and GL vs RL, respectively (Table S10). Among them, most represented TFs were annotated as those encoding C2H2, zn-clus, bZIP, MYB-related, NAC, bHLH, C3H, SNF2, TRAF, WRKY, C2C2-GATA and MYB in the GL vs RL group. The most abundant TFs in GL vs HRL (46 up-regulated and 78 down-regulated) were C2H2, NAC, bHLH, MYB-related, C3H, B3-ARF, WRKY and MYB TFs. In the HRL vs RL comparison (9 up-regulated and 151 down-regulated), C2H2, AP2/ERF-ERF, bHLH, bZIP, C2C2-GATA, C3H, HMG, MYB and MYB-related TFs were the most annotated. Previous studies have shown that transcription factors, such as MYB, bHLH and bZIP, have a deeper effect on anthocyanin biosynthesis^32,33^, and we also found them in many transcription factors in *A. mandshuricum*. For example, most of the MYB TFs (such as Cluster-20561.80755, Cluster-20561.81808, Cluster-20561.110291, Cluster-20561.85332 and Cluster-20561.98223) were co-upregulated with most of the key genes in flavonoid biosynthesis during the discoloration of the leaves in *A. mandshuricum*, and the similar co-upregulated phenomenon was also found in bHLH (Cluster-20561.117348, Cluster-20561.26518, Clus-ter-20561.46232, Cluster-20561.92650, Cluster-20561.107315, and more) and bZIP (Cluster-20561.89129, Clus-ter-20561.98504, Cluster-20561.102544, Cluster-20561.141195, Cluster-20561.92791 and so on) TFs, indicating that those MYBs, bHLHs and bZIPs were important positive regulators in leaf color change periods of *A. mandshuricum*. In addition, it is worth noting that in the transcription factors of the three comparison groups, most bHLHs and bZIPs were downregulated when the leaves changed from green to red, indicating that some of the down-regulated genes mediated negative regulation in *A. mandshuricum* anthocyanin biosynthesis (Table S9).

### Conjoint analysis between transcriptome and metabolome

To better understand the relationship between genes and metabolites, we mapped the DEGs and DAMs to the KEGG pathmap at the same time. From the results, many DEGs and DAMs were significantly enriched in the anthocyanin biosynthesis pathway. At the same time, we selected those results in which the Pearson correlation coefficient was greater than 0.8. A total of 14,310 DEGs associated with 26 metabolites were found, and most of them jointly regulated the synthesis of one or more metabolites. They were significantly associated with Cya-3,5-O-diglu (6696 genes), Cya-3-O-(6-O-malonyl)-glu (6807 genes), Cya-3-O-ara (6884 genes), Cya-3-O-glu (6568 genes), Cya-3-O-rut (6872 genes), Del-3-O-rut (3789 genes), fla_dihydrokaempferol (6622 genes), fla_Quercetin-glu (6010 genes), Mal-3-O-(6-O-malonyl)-glu (3199 genes), Pel-3,5-O-diglu (6899 genes), Pel-3-O-(6-O-malonyl)-glu (6769 genes), Pel-3-O-glu (6821 genes), Peo-3,5-O-diglu (6755 genes), Peo-3-O-glu (6827 genes), Pet-3-O-glu (6536 genes) and others (Table S11).

From the results of Conjoint analysis between these DEGs and their correlated metabolites, we found two key structural genes *ANS* (*Cluster-20561.86285*) and *BZ1* (*Cluster-20561.99238*) in anthocyanidin biosynthesis pathway were significantly up-regulated when the leaves change to red, indicating that they might enhance accumulation of the predominant metabolite cyanidin 3-O-glucoside which is their downstream metabolite, and contributed the red formation of *A. mandshuricum* leaves (Fig. 6).

### qRT-PCR validation of DEGs in transcriptome data

**Figure 8:**
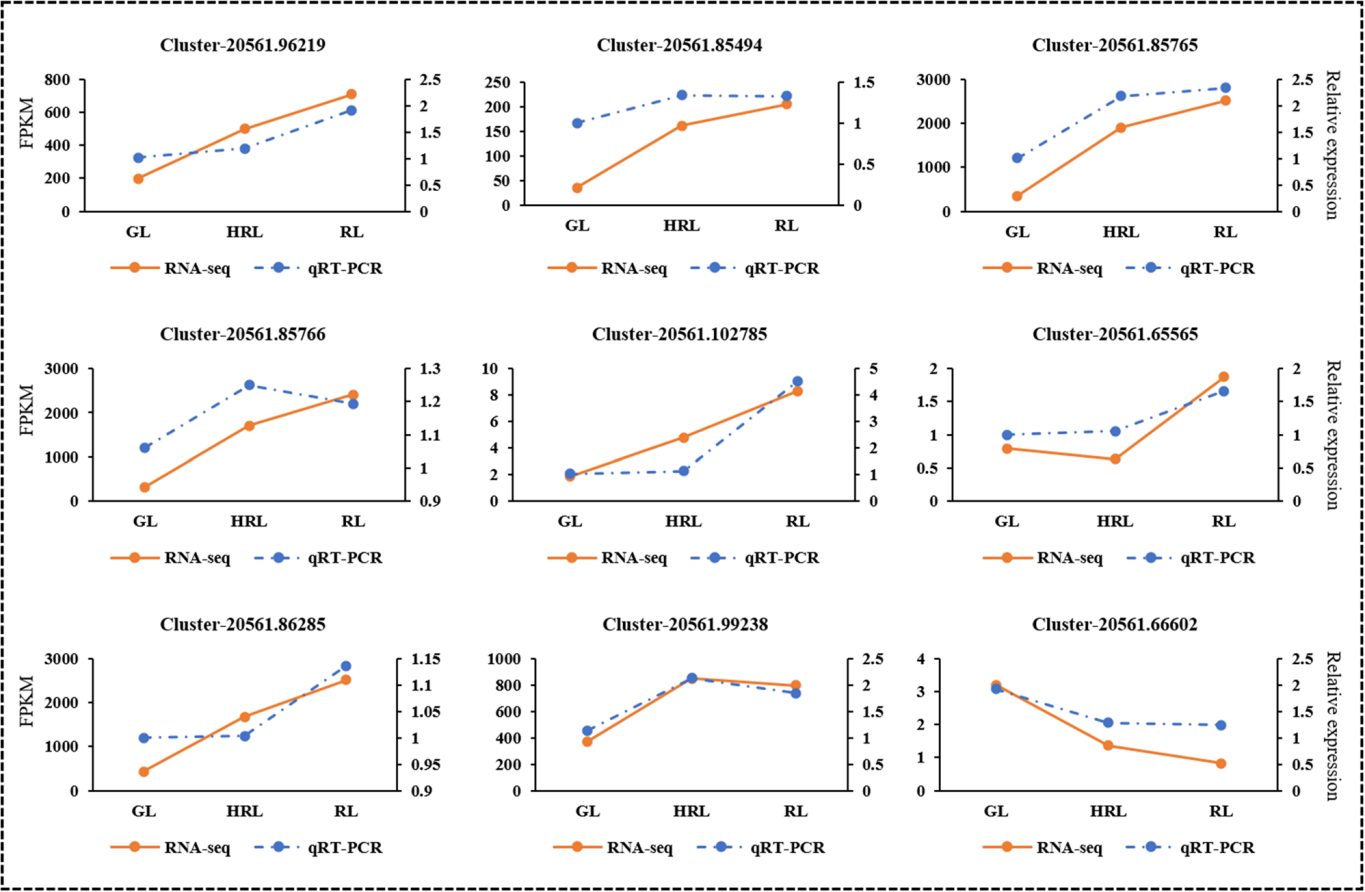
Verification of phenylpropanoid, flavonoid, and anthocyanidin biosynthesis-related differentially expressed genes (DEGs) by quantitative real-time polymerase chain reaction (qRT-PCR).

To further verify the reliability and accuracy of the transcriptome data, we initially screened 12 DEGs (*Cluster-20561.96219*, *Cluster-20561.85494*, *Cluster-20561.85765*, *Cluster-20561.85766*, *Cluster-20561.102785*, *Cluster-20561.65565*, *Clus-ter-20561.86285*, *Cluster-20561.99238* and *Cluster-20561.66602*) which associated with phenylpropanoid, flavonoid, and anthocyanidin biosynthesis to detect expression levels in GL, HRL and RL with qRT-PCR (The primer sequence is in Table S12). The relative expression trends of these candidate genes were similar to those of RNA-seq, indicating the high reliability and accuracy of transcriptome data (Fig. 8). In line with our expectations, the most pivotal genes *ANS* (*Cluster-20561.86285*) and *BZ1* (*Cluster-20561.99238)* in anthocyanin biosynthesis of *A. mandshuricum*, were highly expressed when leaves change from green to red, suggesting that they are the candidate key genes underlying the leaf color formation in *A. mandshuricum*.

## Discussion

*Acer mandshuricum* is of vital ornamental and economic value because of its beautiful colored leaves. Previous studies have shown that the main substances in *A. mandshuricum* red leaf extract pigments are anthocyanins and flavonoids^2^. In our present study, using UPLC and a widely targeted metabolomics approach, we investigated the changes in metabolites in three different colored leaf samples of *A. mandshuricum*, aiming to provide a more comprehensive understanding of the accumulation dynamics of anthocyanins in the process of leaf color change. From the results (Table S1), a total of 26 anthocyanidins were simultaneously detected and quantified, mainly cyanidins, pelargonidins, peonidins, malvidins, delphinidins, petunidins, proanthocyanidins and flavonoids, which showed that they played an important role in the critical period of leaf color change. Furthermore, 17 metabolites, including cya-3-O-glu, cya-3,5-O-diglu, pel-3-O-glu, pet-3-O-glu and others, were up-regulated when the leaves changed from green to red. Most of them have been reported in other plant coloration studies^23,34,35^, indicating that these compounds might be responsible for plants turning red. Especially the metabolite cyanidin 3-O-glucoside was found that significantly correlated with the color formation, was the predominant metabolite in anthocyanin biosynthesis of *A. mandshuricum*, and accumulated in large quantities during the plant red period and was associated with numerous DEGs (6568 genes) (R2 > 0.8) (Tables S1, S10). Therefore, this metabolite, cyanidin 3-O-glucoside, was considered an anthocyanin related to red pigmentation in *A. mandshuricum*. Interestingly, similar results of cyanidin 3-O-glucoside were also reported in some plants with red leaves or fruit^36–38^.

The biosynthesis of anthocyanin metabolites is tightly controlled by some structural genes (i.e., *PAL*, *CHI*, *DFR*, *ANS* and more), which are also regulated by some transcription factors (i.e., MYB, bHLH, and WD40)^39,40^. In the whole biosynthesis of anthocyanin metabolites, early biosynthetic genes (*CHS*, *CHI*, *F3H*, etc.) lead to the production of flavonoids, while late biosynthetic genes (i.e., *DFR*, *ANS*) lead to the production of anthocyanins^41^. We revealed that most of the structural genes that regulated the phenylpropanoid, flavonoid, and anthocyanidin biosynthetic pathways in the leaf’s change to red were highly active (Fig. 6). The expression levels of most genes encoding *PAL*, *C4H*, *4CL*, *CHS*, *CHI*, *F3H*, *DFR*, *F3′5′H*, *ANS* and *BZ1* were higher in RL than in GL, resulting in higher anthocyanin accumulation during the red leaf stage. *F3′H* and *F3′5′H* enzymes are important for anthocyanin accumulation, which could determine the B-ring hydroxylation pattern of anthocyanins^42^, and *ANS* and *DFR* genes have been proven to catalyze the conversion of colorless leucoanthocyanidins into available colored anthocyanidins^43^. In this study, we found that six *F3H* genes (*Cluster-20561.126021*, *Cluster-20561.93983*, *Cluster-20561.93984*, *Cluster-20561.98637*, *Cluster-20561.98639*, *Cluster-20561.98643*), one *F3′5’H* gene (*Cluster-20561.65565*), nine *DFR* genes (*Cluster-20561.102785*, *Cluster-20561.92388*, *Cluster-20561.92390*, *Cluster-20561.92391*, *Cluster-20561.92394*, *Cluster-20561.92503*, *Cluster-20561.97639*, *Cluster-20561.97640*, *Cluster-20561.97641*) and two *ANS* genes (*Cluster-20561.86283* and *Cluster-20561.86285*) were significantly up-regulated in RL, suggesting that they might enhance the red color accumulation, forming leucoanthocyanidin and transferring into anthocyanidins in *A. mandshuricum*. Previous studies have shown that the *ANS* gene is abundantly expressed in cell cultures of red cell lines and seedlings in the rare medicinal plant *Saussurea medusa*^44^, and the high expression of *ANS* and *DFR* also promotes anthocyanin biosynthesis in red pear fruit skin^45^; similar findings have been validated in *Litchi chinensis* Sonn^46^ and *Malus hupehensis*^39^. In addition, the anthocyanidin 3-O-glucosyltransferase (*BZ1*, EC: 2.4.1.115) is the last key enzyme in the anthocyanin biosynthetic pathway and can catalyze the transformation of unstable anthocyanidin into anthocyanin, including by catalyzing the transfer of glucose from UDP-glucose to anthocyanidins including delphinidin, cyanidin, etc.^47–49^. As the leaves changed from green to red, two *BZ1* genes were down-regulated, two genes were up-regulated, and one gene was up-regulated only in half-red leaves. Notably, the *BZ1* gene *Cluster-20561.99238* (Table S8) was highly expressed, and its downstream metabolites (cyanidin 3-O-glucoside, pelargonidin 3-O-glucoside, and delphinidin 3-O-glucoside) were correspondingly significantly upregulated in the RL period (Fig. 6). Therefore, we suggested that *BZ1* gene was the key gene in regulating anthocyanin synthesis and leaf color stabilization. Interestingly, we found that the *UGT75C1* gene (anthocyanidin 3-O-glucoside 5-O-glucosyltransferase, EC:2.4.1.298) was significantly down-regulated in the anthocyanin synthesis process, leading to the formation of more derivatives of anthocyanins.

Some TFs, such as MYB, bHLH, and WD40, are key regulatory factors for structural genes in flavonoid biosynthesis pathways^50,51^. The MYB transcription factor family is very large and versatile in plants, and these TFs can regulate the biosynthesis of phenylpropanoids^52^. Therefore, many MYB genes were identified, which were considered potential key regulators in the anthocyanin biosynthesis pathway^53^. MYB, bHLH, and WD40 can be combined into the transcription factor complex MBW (MYB-bHLH-WD40) to regulate the biosynthesis of anthocyanins^54^; they have been shown to regulate the synthesis of anthocyanins positively or negatively in plants including banana^55^, rice^56^, persimmon^57^, etc. In the present study, a total of 341 (106 up-regulated and 235 down-regulated), 454 (103 up-regulated and 351 down-regulated) and 981 (346 up-regulated and 635 down-regulated) differentially expressed TFs were identified in GL vs HRL, HRL vs RL and GL vs RL, respectively (Table S9). Among them, we identified several candidate MYB (*Cluster-20561.80755*, *Cluster-20561.81808*, *Cluster-20561.96473*, and *Cluster-20561.98223*) and bHLH (*Cluster-20561.92650*) TFs that were highly up-regulated with most of the key genes in flavonoid biosynthesis during the discoloration of the leaves in *A. mandshuricum* and may positively regulate the key structural genes in anthocyanin synthesis in *A. mandshuricum* (Table S8). In addition, some TFs such as NAC, RWP-RK. WRKY, C2H2, GARP-G2-like, MYB-related, HD-Zip, and bZIP were discovered during the leaf color change periods and displayed different expression levels, indicating that they may co-regulate the expression level of structural genes involved in the synthesis of flavonoids, similar to the results of recent studies^58,59^.

## Materials and methods

### Plant materials

Fresh plant leaves of *A. mandshuricum* were harvested during three consecutive leaf color change periods, i.e., green leaf (GL), half red leaf (HRL) (During this period, the leaves began to change from green to red, and clearly visible red pigment began to appear on the leaves), and red leaf (RL) (Fig. 1). All samples were taken in the year 2020, green leaves were harvested at 10 a.m. on June 15, half red leaves were harvested at 10 a.m. on September 22, and red leaves were harvested at 10 a.m. on October 4. The leaves of all three periods were harvested from the same tree. The samples were frozen in liquid nitrogen and stored at − 80 °C until further use. Each color leaf contains three biological repeats. For each repeat, normal growth and no pests or diseased leaves were considered for one mixed sample^34^, and the total sample weight was more than 3 g. These samples were used for subsequent transcriptome and metabolome studies.

Fresh samples were collected at the Northeast Forestry University (North: 45.723999; East: 126.635474) in Heilongjiang Province, China. And the experimental research on the plants described in this study comply with institutional, national and international guidelines.

### Sample preparation and metabolite extraction

For metabolite extraction, five steps needed to be completed. (1) The samples were subjected to freeze-dry. (2) A mixer mill (MM 400, Retsch) was used to crush the freeze-dried samples with a Zirconia bead for 1.5 min at 30 Hz. (3) 50 mg powder was weighed and extracted with 0.5 ml methanol/water/hydrochloric acid (799:200:1, V/V/V). (4) The extractives were vortexed for 10 min, ultrasonicated for 10 min, and centrifuged at 12,000×*g* under 4 °C for 3 min, absorbed the supernatants. The step 4 repeated twice. (5) The supernatants were collected twice and filtered (PTFE, 0.22 μm; Anpel) before LC–MS/MS (Liquid Chromatography-Tandem Mass Spectrometry, https://sciex.com.cn) analysis.

### Metabolite analysis

Professional analytical processes of LC–MS/MS were performed by MetWare (http://www.metware.cn/), using the same methods described in previous articles^60,61^. Metabolite data analysis was conducted with the R Programming language (https://www.r-project.org/), and hierarchical cluster analysis (HCA) and orthogonal partial least-squares discriminant analysis (OPLS-DA) were performed to identify the accumulation pattern in metabolites from nine leaf samples of three colors. The OPLS-DA model was used to identify the differences in metabolite composition between the samples by variable importance in projection (VIP) values (VIP ≥ 1) and Fold change ≥ 2 or ≤ 0.5.

### RNA extraction, quantification and sequencing

RNA extraction, quantification and transcriptome sequencing were performed as fully described in Lu’s study^62^. For transcriptome sequencing, we constructed nine libraries representing the three leaf stages with three biologically replicates for each period. All libraries were sequenced on an Illumina HiSeq platform, and raw data with 150 bp paired-end reads were obtained for subsequent analysis.

### Transcriptome data analysis

The raw data (or raw reads) were filtered with fastq software (https://www.bioinformatics.babraham.ac.uk/projects/fastqc/) to filter out adapters and low-quality sequences and obtain high-quality clean sequences. Then, we used Trinity software (version 2.6.6) to perform De novo assembly. To obtain the annotation information, the unigenes were compared with public databases, including KEGG^63,64^, GO, KOG, NR, Swiss-Prot, Pfam and Trembl. FPKM (fragments per kilobase of transcript per million fragments mapped) was used as an indicator to measure the level of transcript or gene expression. Differential expression analysis between different comparisons of colored samples was performed using the DESeq2 R package (1.20.0)^65^. When the difference analysis was completed, the Benjamini–Hochberg method was used to carry out multiple hypothesis test corrections for the P value to obtain the false discovery rate (FDR). The screening conditions of unigenes were |log_2_(fold change)|≥ 1 and FDR < 0.05^66^. KEGG and GO enrichment analyses of differentially expressed genes were further implemented employing the KOBAS 2.0 software^67^ and GOseq R packages^68^, respectively.

### Gene validation by real-time quantitative PCR (qRT-PCR)

To further verify the reliability and accuracy of the transcriptome data, we initially screened 12 DEGs associated with phenylpropanoid, flavonoid, and anthocyanidin biosynthesis to detect expression levels in GL, HRL and RL with qRT-PCR. The Tiangen total RNA extraction kit (Tiangen, Beijing, China) and PrimeScript RT reagent Kit with gDNA Eraser (TaKaRa, Kyoto, Japan) were used for RNA extraction and cDNA synthesis, respectively. Primer 3 software (version 2.5.0, https://primer3.ut.ee/) was used to design specific primers for qRT-PCR analysis. qRT-PCR was carried out with the TaKaRa SYBR Green Mix kit (TaKaRa, Beijing, China) using an ABI 7500 Fast Real-Time Detection System. The PCR amplification experiment was performed with a total volume of 20 μl (10 μl 2xSYBR Primix ExTaq, 0.4 μl ROX-reference, 6 μl ddH_2_O, 0.8 μl downstream primer, 0.8 μl upstream primer, and 2 μl cDNA template under a standard program (95 °C for 30 s, 40 cycles at 95 °C for 5 s, 60 °C for 35 s, and 95 °C for 15 s, and 60 °C for 1 min, followed by 95 °C for 15 s). The relative expression analysis of quantitative data was performed using the 2^−ΔΔCT^ method^69^ with reference gene 18S-RNA as a control.

## Conclusion

In this study, the molecular mechanisms of anthocyanin biosynthesis in *A. mandshuricum* during different leaf color change periods were revealed based on metabolomics and transcriptomics studies. We analyzed the accumulation pattern of anthocyanins in three different leaf color states and found that cyanidin 3-O-glucoside was the predominant metabolite in the anthocyanin biosynthesis pathway, which accumulated in large quantities during the red leaf period. Furthermore, we analyzed the differentially expressed genes associated with anthocyanin biosynthesis in the leaves at different times. A variety of regulatory models of structural genes (*PAL*, *ANS*, *BZ1*, etc.) and some potential transcription factors (MYB, bHLH, C2H2, NAC, bZIP, etc.) involved in biosynthesis of flavonoid pathways and anthocyanin biosynthesis pathway were found. The regulation of these key structural genes or their transcription factors might help elucidate the mechanism of leaf color change in *A. mandshuricum*. Our studies could provide novel insight into the molecular regulation of anthocyanin biosynthesis and accumulation associated with leaf color change in *A. mandshuricum*.

## Supplementary Information

Supplementary Figures.

Supplementary Tables.

## Data Availability

Transcriptome sequencing data are available in the SRA database of National Center for Biotechnology Information (NCBI) under the accession number of PRJNA741917.
