# Supplementary material for: Combined transcriptome and metabolome integrated analysis of Acer mandshuricum to reveal candidate genes involved in anthocyanin accumulation
Source: Sci Rep. 2021 Nov 30;11:23148. doi: 10.1038/s41598-021-02607-2 (PMC8633053; doi:10.1038/s41598-021-02607-2)
Supplement: Supplementary file 1 — Supplementary Figures. [file 41598_2021_2607_MOESM1_ESM.pdf]

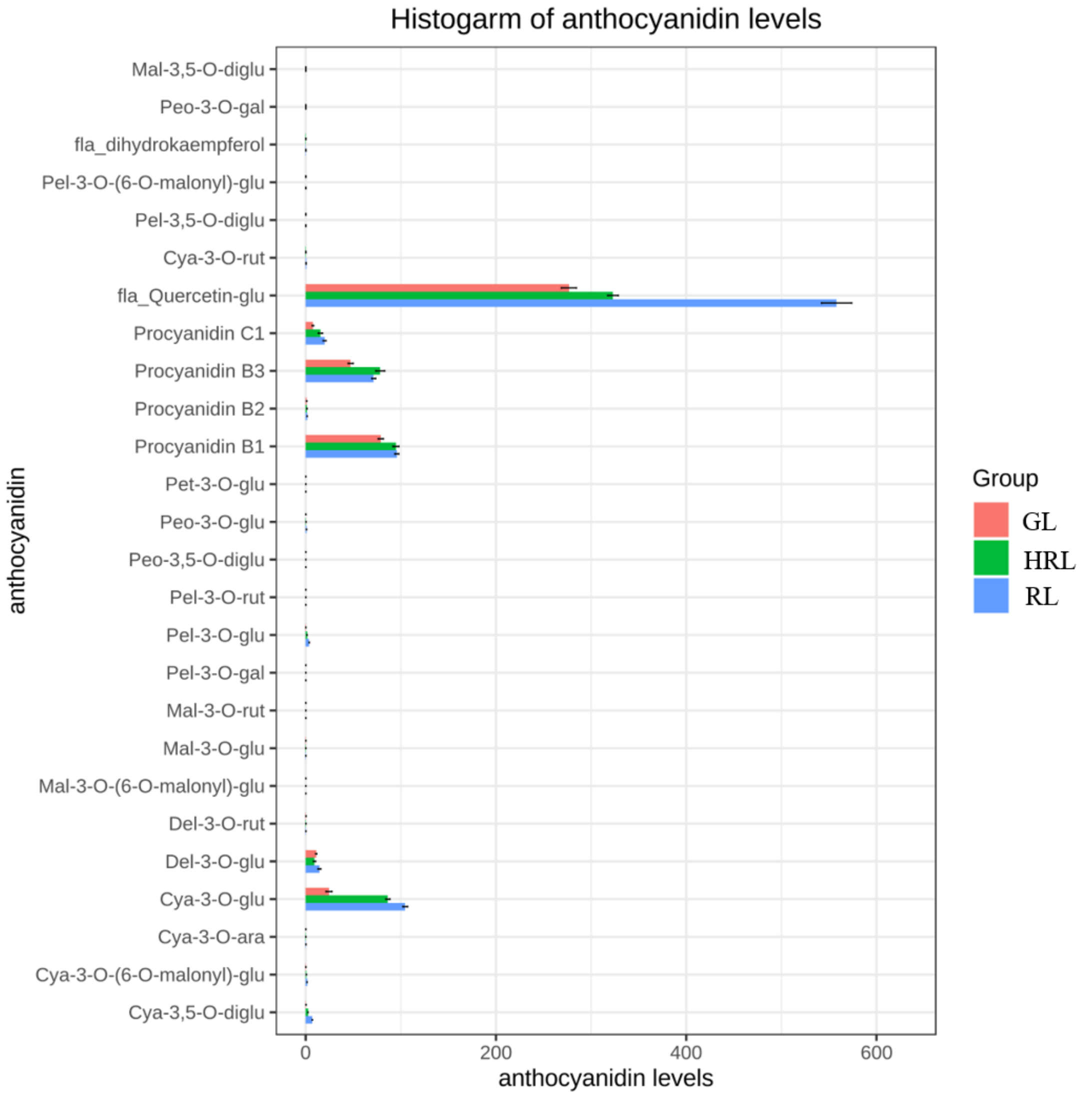

Figure S1. Anthocyanidin levels of *A. mandshuricum*

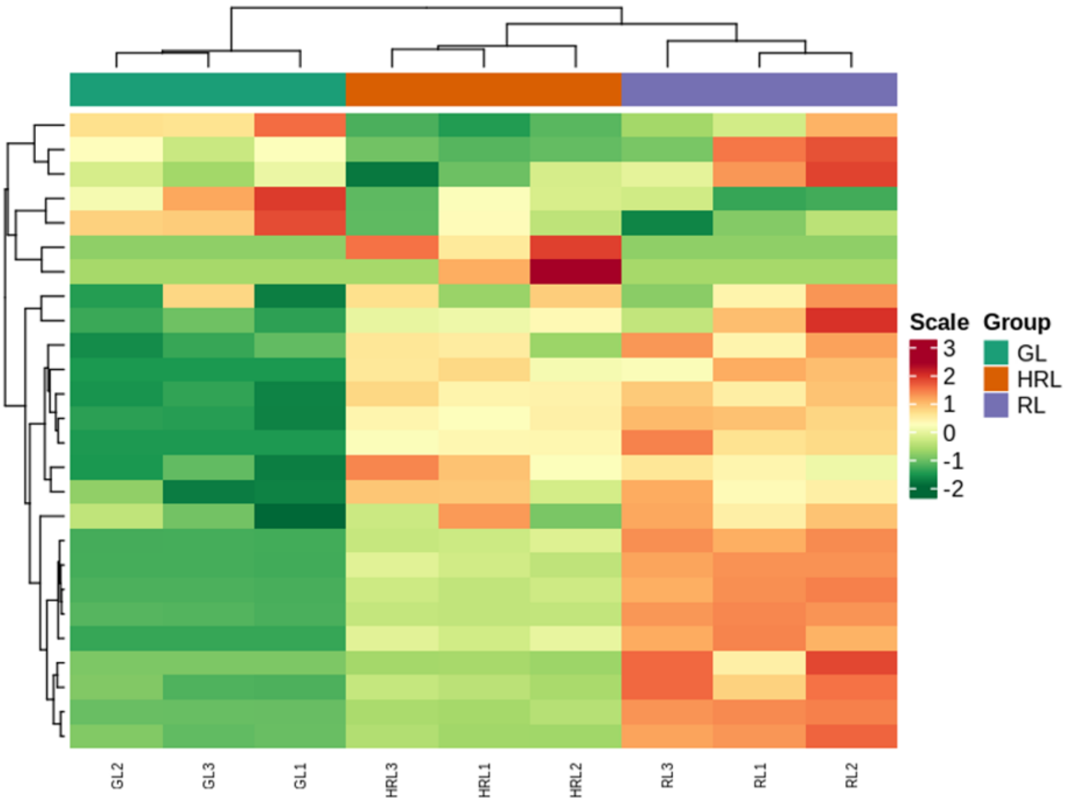

Figure S2. Cluster heatmap of anthocyanins in *A. mandshuricum*

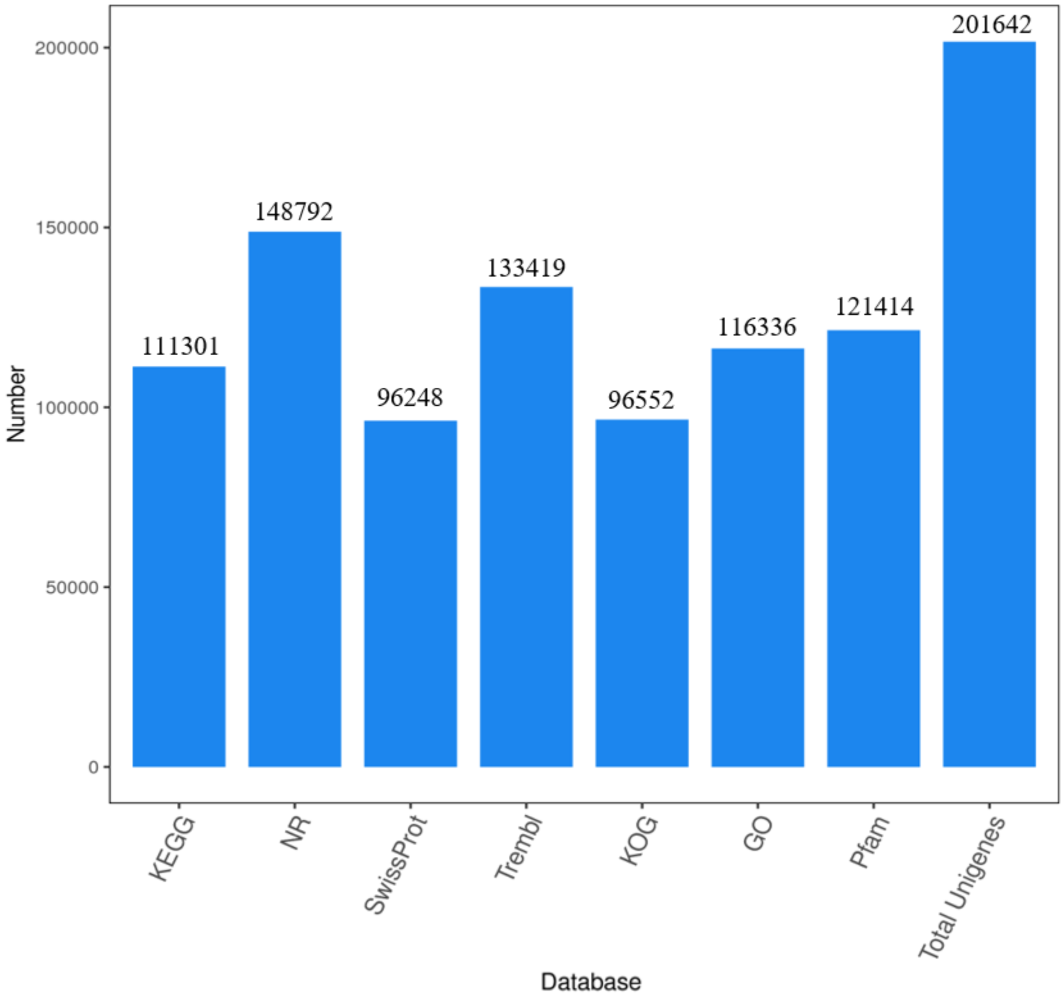

Figure S3. Annotation statistics of Unigenes

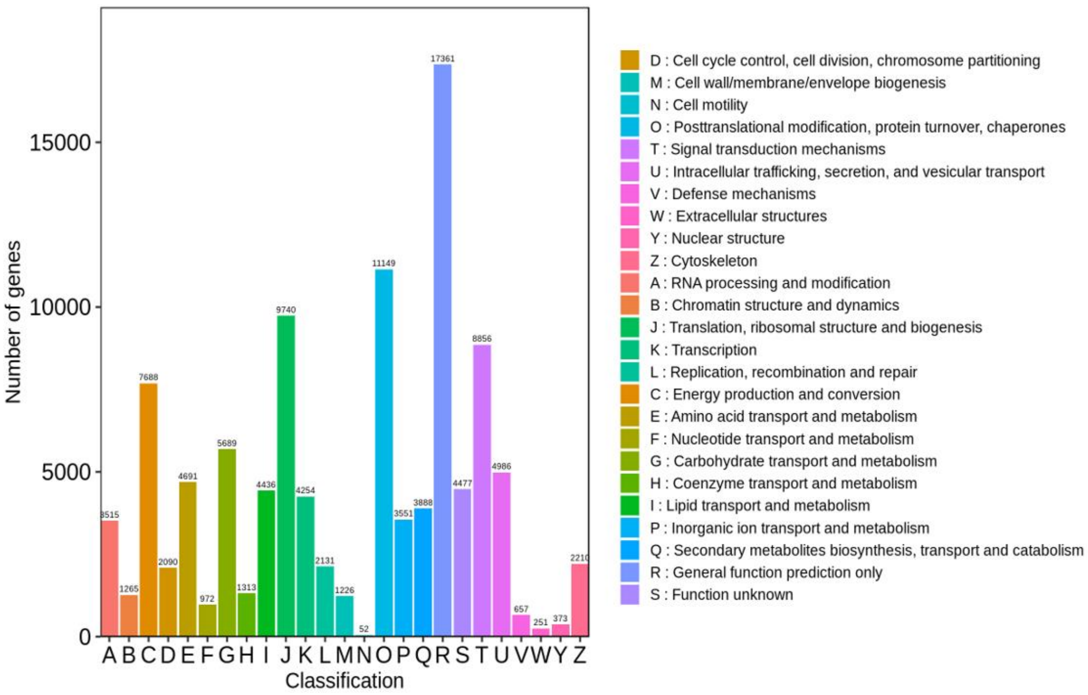

Figure S4. Annotations of KOG database

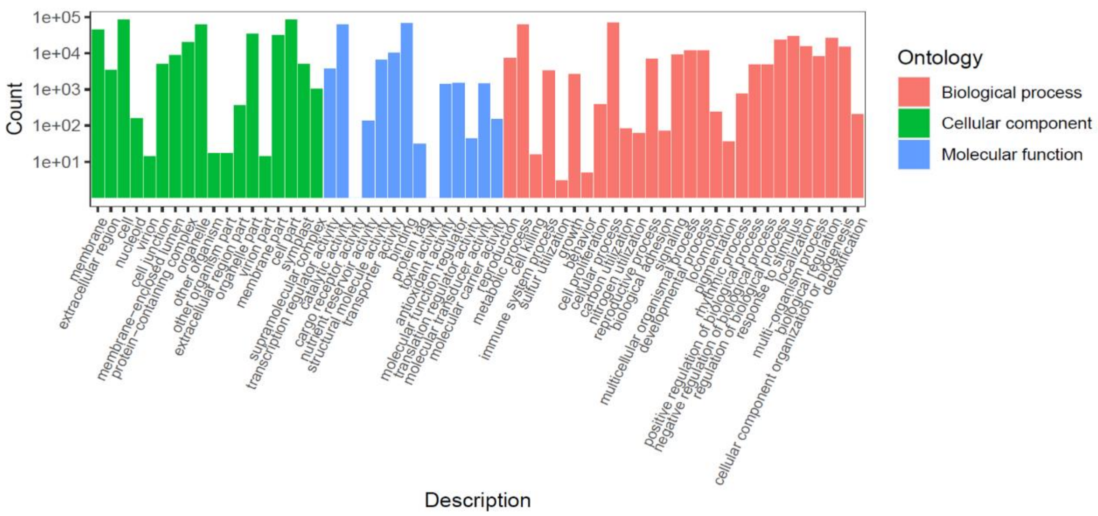

Figure S5. Annotations of GO database

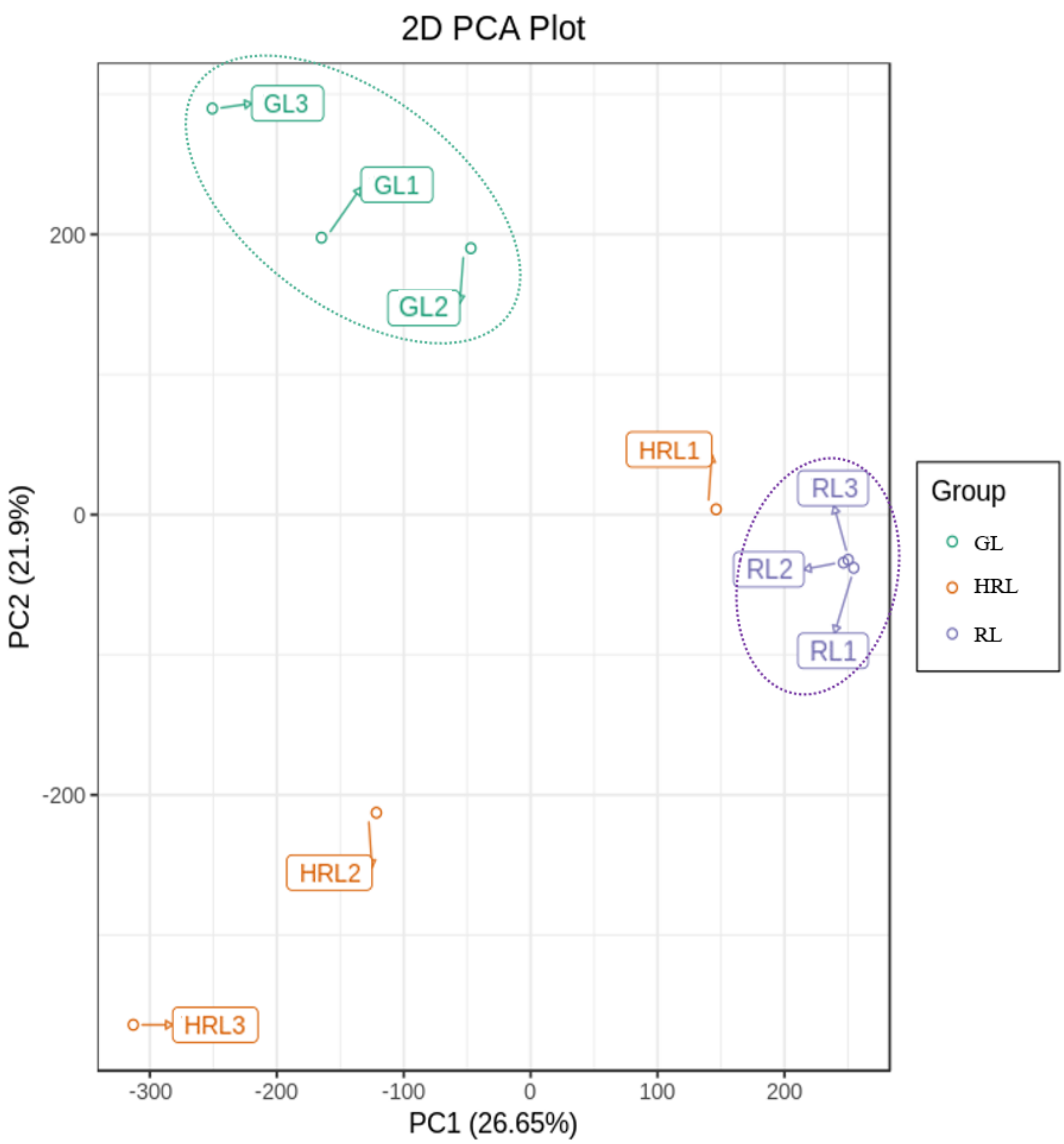

Figure S6. Figure of principal component analysis in samples

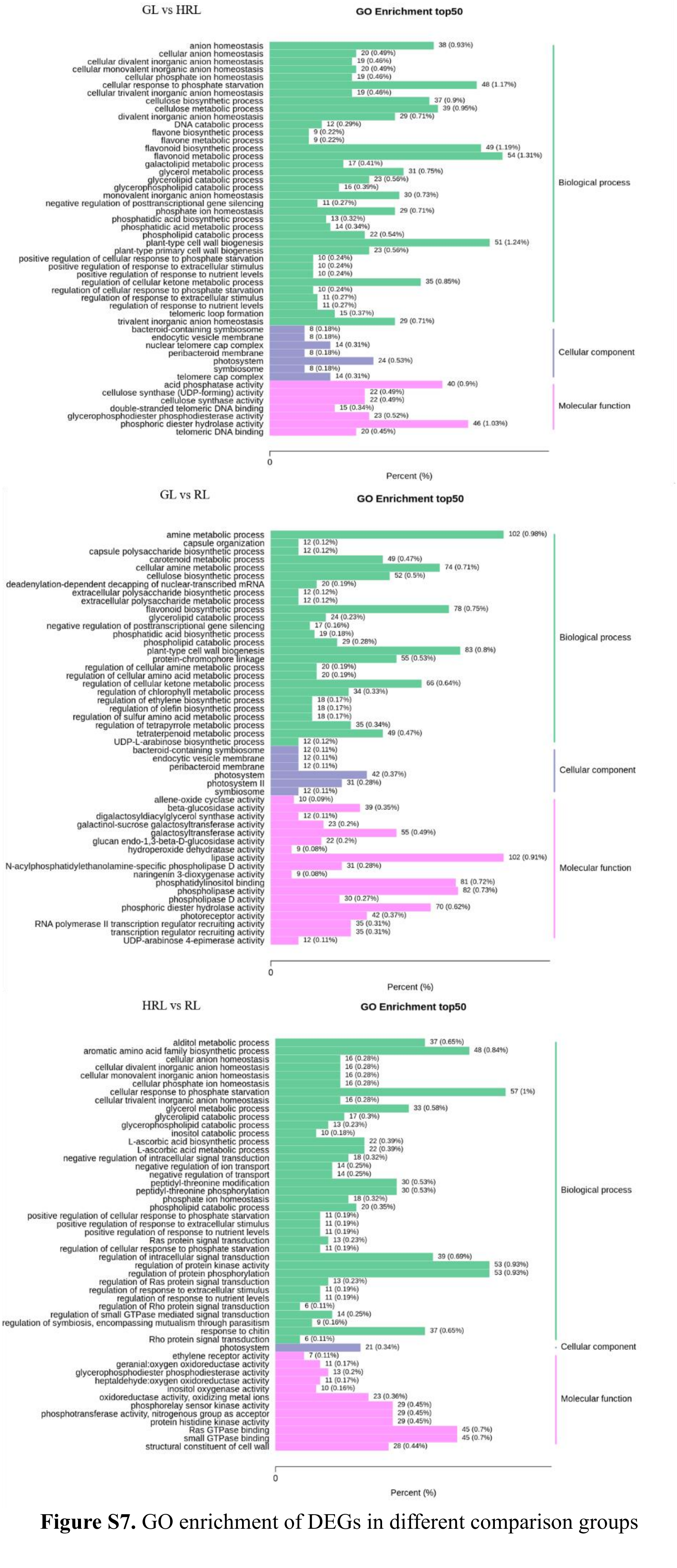

Figure S7. GO enrichment of DEGs in different comparison groups

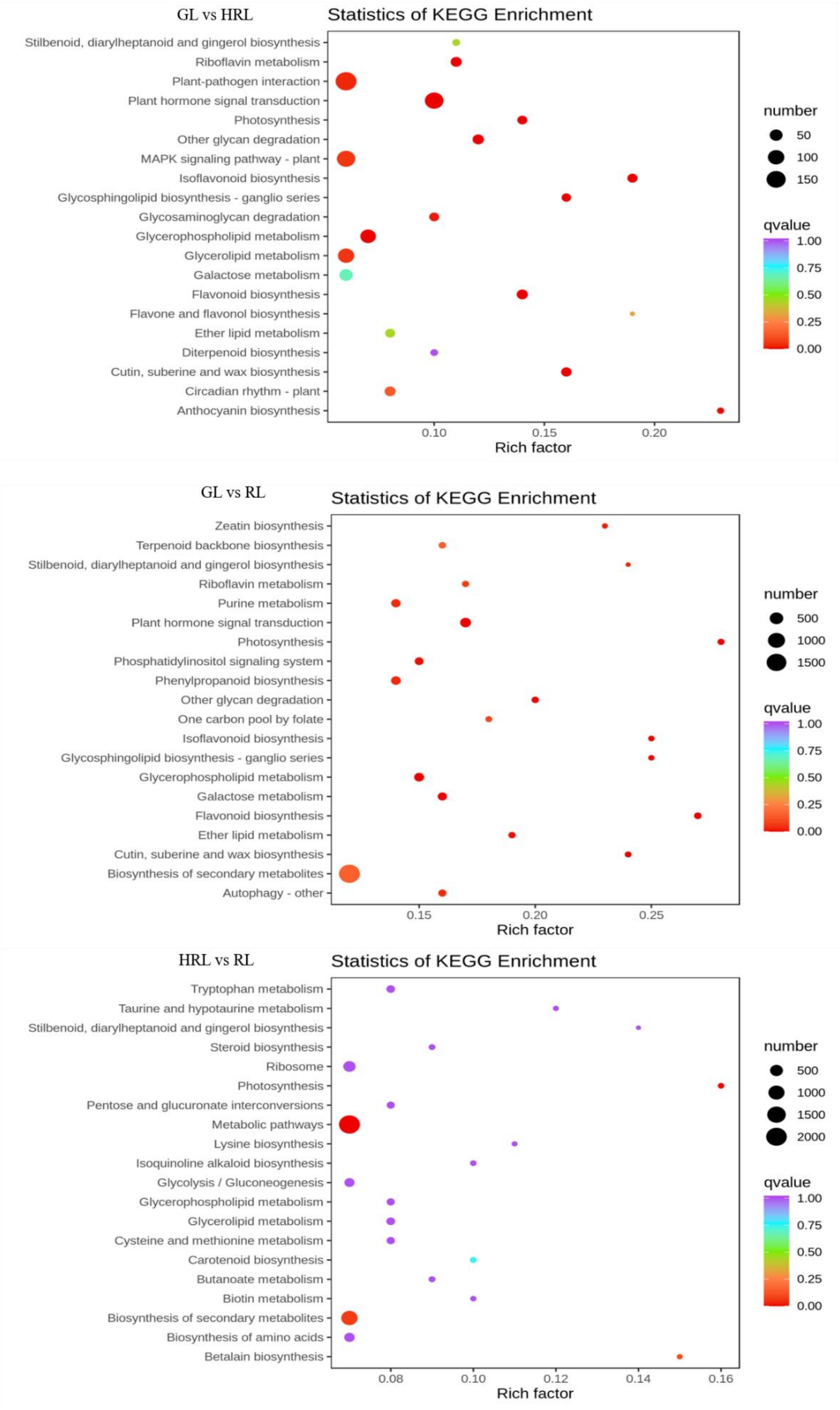

Figure S8. KEGG enrichment of DEGs in different comparison groups
